# Metastatic Vulval Crohn’s Disease With Good Outcome on Ustekinumab

**DOI:** 10.7759/cureus.16252

**Published:** 2021-07-08

**Authors:** Yizhe Lim, Manpreet Singh

**Affiliations:** 1 Obstetrics and Gynaecology, Torbay Hospital, Torquay, GBR

**Keywords:** vulvar, metastatic crohn’s disease, crohn’s disease (cd), ustekinumab, gynaecology, gastroenterology, vulval, cutaneous crohn’s disease

## Abstract

Vulval Crohn’s disease is a rare manifestation of Crohn’s disease. Although it is usually caused by a fistulating process, it may be a result of a metastatic disease process as well, the exact mechanisms of which are not well understood yet. Vulval Crohn’s disease may occur before the onset of bowel symptoms and may continue to be active while bowel symptoms are quiescent. Coupled with the fact that most vulval Crohn’s lesions are similar to other disease processes, it can prove to be a diagnostic challenge. Due to its rarity, no guidelines for treatment exist; however, most reports agree that when symptoms are not controlled with antibiotics and topical steroids, immunosuppressive medications such as oral steroids and biological agents are the way forward. Although surgery may be an option, data suggest that its use is scarce. We report the case of a 47-year-old Caucasian woman with bilateral metastatic vulval Crohn’s disease from a district hospital in the United Kingdom who responded well to ustekinumab. This is one of the first few cases reporting good clinical outcomes with the agent.

## Introduction

Crohn’s disease is a type of inflammatory bowel disease of unknown cause and features chronic inflammation and ulcers most commonly in the bowel. It is thought to affect 10 in every 100,000 people each year. It can cause fistulas to form between the bowel, vagina, bladder, rectum, and skin. Genital Crohn’s disease is a rare extraintestinal manifestation of Crohn’s disease. Most lesions are caused by a fistulating process; however, chronic inflammatory deposits can be found on the skin and mucosa anywhere in the body, without a fistula, and independent of bowel disease. This condition is termed metastatic Crohn’s disease [1]. The ulcerations in the genitals in very rare cases may be caused by the extension of pyoderma gangrenosum, another cutaneous manifestation of Crohn’s disease [2].

Most cases of vulval Crohn’s disease tend to be unilateral, with the most common complaints initially being swelling, ulcers, pain, and itching [3]. This is thought to be due to lymphatic obstruction by the granulomas, which if untreated can lead to further skin breakdown and ulceration [4]. This can be easily overlooked due to its non-specific nature, and genital Crohn’s disease can occur without or even before bowel involvement in up to 35% of cases [5]. Other signs include erythema, abscess, discharge, and mass [3].

The differential diagnoses are wide-ranging, with a fluctuant mass being often misdiagnosed as Bartholin’s cyst or abscess. It can also be due to atypical infections, such as tuberculosis, actinomycosis, lymphogranuloma venereum, and genital herpes. It can also be a part of hidradenitis suppurativa, intertrigo, and sarcoidosis [6]. However, malignancy, whether primary or secondary metastatic deposits, should always be considered as these patients are at risk for squamous cell carcinomas [7], especially if the patient is on immunosuppressants, as in our case. The appearances of the conditions are often very similar and can pose a diagnostic challenge if a high index of suspicion is not present.

## Case presentation

A 47-year-old Caucasian woman presented to the gynaecology outpatient department following a referral by her general practitioner (GP) for suspected vulval cancer. She reported bilateral swelling of her labia and vulva with itching, pain, and ulceration for a year. She had several courses of antibiotics, antihistamines, and topical antifungal therapy with the GP to no avail. She was being treated for Crohn’s disease with immunosuppressive agents including biologics a year ago, which prompted the current referral.

The examination performed in the clinic revealed bilateral swelling and shallow fissures of her vulva and labia. There was also superficial ulceration. The left side exhibited more erythema, some purulent discharge, and friable-appearing tissue, consistent with secondary infection. An internal examination did not reveal any fistula. There were no palpable inguinal lymph nodes, and the remainder of her pelvic examination was reassuring. The lesions were atypical of infection alone, and with the clinical question of vulval cancer, the lesions were biopsied and sent for histopathological examination.

**Figure 1:**
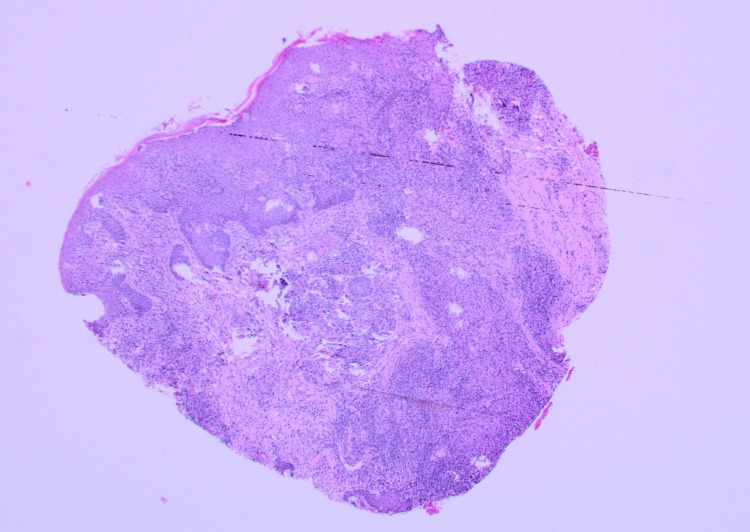
Low-power view showing the entire specimen with dense inflammation.

**Figure 2:**
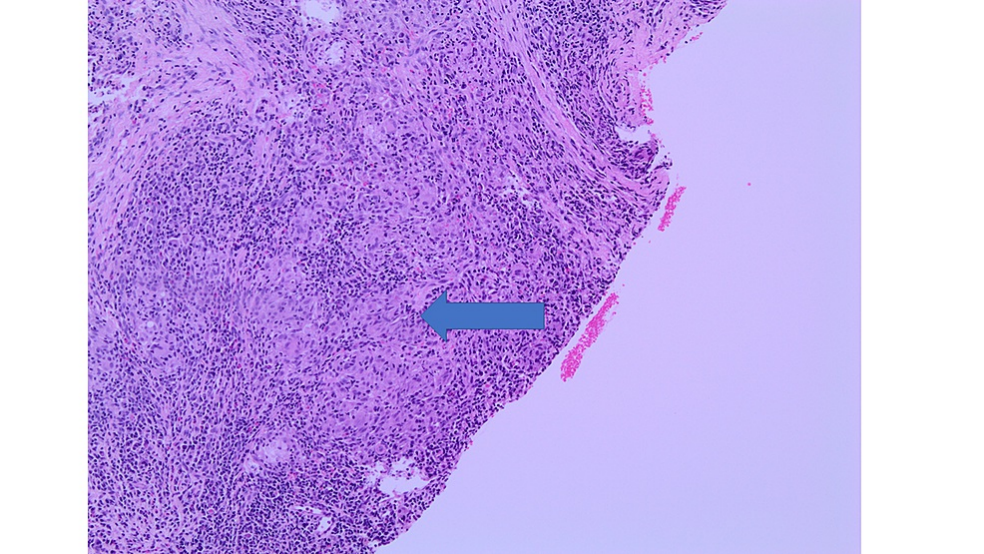
Medium-power view showing an area of granuloma with loose appearance and surrounding dense chronic inflammation (labelled by an arrow).

**Figure 3:**
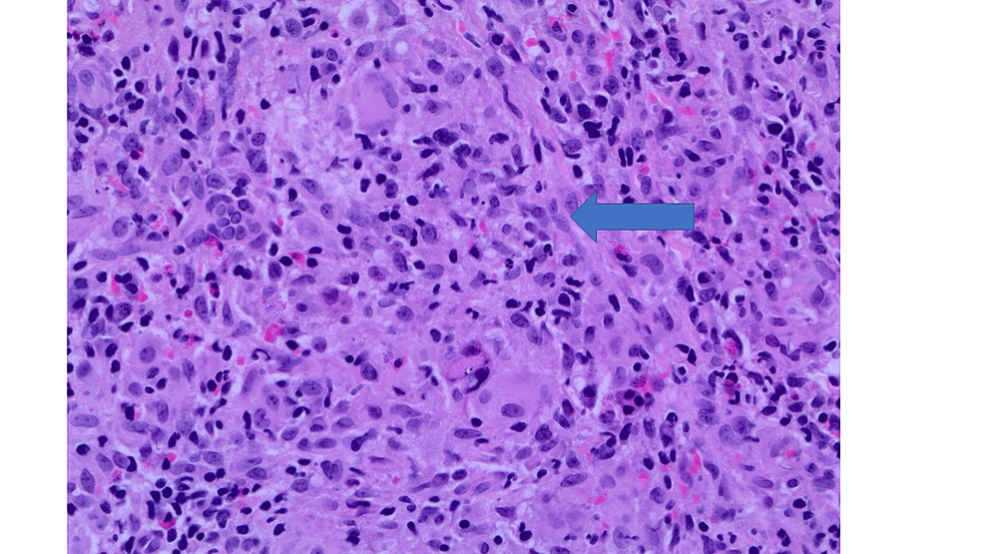
High-power view showing the details of individual histiocytes within the granuloma without evidence of central caseation (labelled by an arrow).

The biopsy results showed non-caseating granulomas and dense chronic inflammation, without fungal hyphae or acid-fast bacilli (Figures 1-3). In addition, no malignant cells were seen. The histology samples were compared with her Crohn’s samples and were found to be consistent. In the absence of fistulas, the final diagnosis of bilateral metastatic Crohn’s disease of the vulva was made.

Initial treatment with oral metronidazole was started as empirical treatment for secondary infection. The patient reported some clinical improvement but the lesions remained bothersome. Her case was discussed with the gastroenterologist responsible for her care of Crohn’s disease and it was decided to reinstate her biological therapy. However, due to a long drug holiday, her antibodies needed to be checked before restarting therapy, and it was found that she had developed antibodies against infliximab. The decision was made to start ustekinumab instead, after which the patient reported excellent and rapid recovery of the vulval lesions.

She was brought back to the clinic after six months, and after noting a near-full recovery, she was discharged from gynaecology.

## Discussion

Most studies suggest that a definitive diagnosis of Crohn’s disease is best made with a biopsy of the lesion, which shows non-caseating granulomas and chronic inflammatory change in the background [8]. Some studies have classified lesions with granulomas as vulval granulomatosis in the absence of Crohn’s disease [9]. However, having biopsy-confirmed diagnoses is not agreed upon. A case series from a private vulval centre included patients without histological diagnosis and treated them similarly. However, they reported good outcomes with the treatment [10].

Along with investigation, after conducting baseline blood tests, some studies recommend a chest X-ray and QuantiFERON or tuberculin test to exclude sarcoidosis and tuberculosis as a cause [11]. There may or may not be features of Crohn’s disease present, and if the diagnosis is suspected, a referral to a gastroenterologist should be considered for endoscopy to diagnose intestinal Crohn’s disease [4]. However, isolated cases and independent vulval disease activity does not make this a strict requirement [8,11].

However, when malignancy is suspected, histopathological diagnosis is required for accurate diagnosis, staging, and treatment. Wound swabs for sexually transmitted illnesses and general pathogens, along with analysis of fungi and acid-fast bacilli for biopsy specimens, should be undertaken to rule out any atypical organisms that would be contributing to or causing the skin lesions.

Treatment of vulval Crohn’s disease is not different from standard regimes [3,4,6-8,10,12]. Metronidazole with or without adjunctive steroids has shown to be effective for many cases; however, disease recurrence, with some reporting exacerbation after sudden cessation of treatment, is an issue [8]. Furthermore, long-term metronidazole is associated with peripheral neuropathy, which can limit its continued use [13]. With the advent of biological agents, several studies have reported excellent outcomes with infliximab and azathioprine [3,4,6-15] among others like ustekinumab, which have shown rapid resolution of symptoms similar to the outcome in our patient [15].

When medical therapy fails, surgical treatment may be considered. Surgical approaches usually include incision and drainage of abscesses, local resection of the lesion, and/or a vulvectomy [3]. If an abscess is present, relieving the infective burden is needed, and immunosuppression should be stopped or delayed to prevent the further spread of infection. The outcomes for local excision of lesions are discouraging, likely owing to the nature of Crohn’s disease to recur and poor wound healing [3]. Interestingly, debridement of perianal Crohn’s disease may improve co-existing vulval abscesses [16]. Other methods of surgery such as excision of pedunculated tags and laser treatment for lymphangiectasias have been shown to be beneficial in some patients [3]. Hence, the use of surgery to treat vulval Crohn’s lesions, especially after exhausting other treatment options, should be carefully decided by the clinician and the patient.

## Conclusions

Although vulval Crohn’s disease is primarily caused by a fistulating process, it may be metastatic. It is a rare manifestation of Crohn’s disease that poses diagnostic and management challenges. A high index of suspicion by the clinician is required to provide timely diagnosis and care as it may manifest before any bowel disease is apparent. Aggressive treatment may be required if the diagnosis is confirmed, usually with biological agents as part of Crohn’s, but in some cases, surgical treatment may be indicated.
